# Supplementary material for: Technology empowerment: Digital transformation and enterprise ESG performance—Evidence from China’s manufacturing sector
Source: PLoS One. 2024 Apr 17;19(4):e0302029. doi: 10.1371/journal.pone.0302029 (PMC11023589; doi:10.1371/journal.pone.0302029)
Supplement: Attachment — Submitted filename: Response to Reviewers.docx [file pone.0302029.s003.docx]

Dear Editor and reviewers,

We sincerely appreciate your correspondence and the reviewers' insightful comments on our manuscript titled "Technology Empowerment: Digital Transformation and Enterprise ESG Performance - Evidence from China's Manufacturing Sector" (Manuscript ID: PONE-D-23-39817R1).These comments are all valuable and helpful for improving our article .Revisions were extensively made to the manuscript based on feedback from editors and reviewers. In the revised version, changes have been highlighted in blue.

The main changes made in response to comments from editors and reviewers are summarized below:

**Responds to the Editor's comments:**

**1.**Please review your reference list to ensure that it is complete and correct. If you have cited papers that have been retracted, please include the rationale for doing so in the manuscript text, or remove these references and replace them with relevant current references. Any changes to the reference list should be mentioned in the rebuttal letter that accompanies your revised manuscript. If you need to cite a retracted article, indicate the article’s retracted status in the References list and also include a citation and full reference for the retraction notice.

***Reply:***Thank you very much indeed for your comments.According to your suggestion, we have reorganized the references and replaced the original 7, 11, 22 , 24 and 99.The original reference with serial number 100 was deleted.

**Responds to the reviewer’s comments:**

**Reviewer #1:**

**1.**The firm fixed effects shall be added in the benchmark model, if not, please explain why.

***Reply:***Thank you very much indeed for your comments.In the Benchmark regression analysis, this study builds upon existing research findings and incorporates time fixed effects and industry fixed effects to ensure model stability[1,2]. Additionally, to address potential issues of omitted variables, firm fixed effects, industry fixed effects, and time fixed effects were included in the robustness test. The results remain significant, thus further confirming hypothesis 1 proposed in this study.

The regression results presented in the following table incorporate firm fixed effects, time fixed effects, and industry fixed effects.

|  | (1) |
| --- | --- |
| VARIABLES | ESG |
| Digital | 0.542*** |
|  | (0.148) |
| Size | 1.895*** |
|  | (0.189) |
| Lev | -3.331*** |
|  | (0.718) |
| Grow | -0.336* |
|  | (0.192) |
| Cashflow | 1.508 |
|  | (1.132) |
| Indire | 0.953 |
|  | (1.765) |
| Listage | 2.114 |
|  | (1.404) |
| Top1 | 1.862 |
|  | (1.145) |
| Constant | -23.092*** |
|  | (5.589) |
| Observations | 5,962 |
| R-squared | 0.848 |

Robust standard errors in parentheses

*** p<0.01, ** p<0.05, * p<0.1

**Reviewer #2:**

**1.**Why there is numberings throughout the manuscript. Remove all the numberings in the manuscript.

***Reply:***Thank you very much indeed for your comments.We have removed all numbers from the article.

**2.**Discussion is still lacking its content.

Provide a concise summary of your main findings and interpret the results in the context of your research question or hypothesis. Discuss any unexpected or contradictory findings and offer possible explanations. Compare your findings with previous research in the field. Highlight areas of agreement or divergence between your results and existing literature. Discuss how your findings contribute to, extend, or challenge existing knowledge. Highlight the novelty, significance, or relevance of your findings. Discuss how your study advances knowledge, fills gaps in the literature, or opens up new avenues for research.

***Reply:***Thank you very much indeed for your comments.According to your suggestions, we have made detailed revisions to the discussion section. The specific modifications are as follows:Firstly, the empirical analysis results confirm the hypothesis (H1) proposed in this study. This finding aligns with existing research and further substantiates that digital transformation not only positively impacts financial performance but also serves as an internal driver for enhancing ESG performance within enterprises .

Furthermore, this study confirms the proposition H2. Existing literature predominantly examines the relationship between digital transformation and ESG performance through the lenses of total factor productivity and dynamic capability, neglecting the role of corporate transparency in this context. On one hand, the application of digital technology brings about technological advantages that generate a "governance effect," enhancing internal governance capabilities by increasing shareholder participation in decision-making and curbing managerial discretion. On the other hand, digital transformation yields a "spotlight effect" that amplifies market attention towards enterprises and facilitates greater interaction frequency of internal and external information, thereby promoting environmental and social investments among manufacturing firms to enhance their ESG performance. This research finding expands upon existing knowledge regarding the mechanisms linking digital transformation with enterprise ESG performance while contributing to non-economic value research within the domain of digital transformation.

This study also investigates whether the relationship between digital transformation and ESG performance is influenced by the performance expectation gap, and the empirical findings confirm the hypothesis H3b proposed in this study. The underlying reason is that during a performance expectation gap, enterprises face increased strategic risks and heightened internal and external pressures on management. Pursuing strategic reforms at such times may not yield immediate turnaround results but can potentially lead to organizational difficulties. Consequently, the performance expectation gap tends to foster more conservative strategic decision-making by management, thereby limiting the extent to which digital transformation can promote ESG performance. This conclusion underscores the significance of performance feedback in understanding the intrinsic connection between digital transformation and ESG performance within enterprises while addressing existing research limitations.

**3.**Data analysis was performed using Python and Stata version 16.0 software but it was not stated in the manuscript

***Reply:***Thank you very much indeed for your comments.According to your suggestion, we have included a description of the software used for data analysis in the data sources section.

**4.**Have a title on discussion and discuss thoroughly on whether the objectives achieved or otherwise based on the analysis.

Have a title on conclusion and limitations.

These titles need to be separated.

***Reply:***Thank you very much indeed for your comments. According to your suggestions, we have revised the conclusions, discussions, and limitations of the article. The specific changes have been highlighted in blue in the revised manuscript.

According to the editor and reviewers’ comments, we have made extensive modifications to our manuscript and supplemented extra data to make our results convincing. Thank you again for your positive comments and valuable suggestions to improve the quality of our manuscript.If there are any other modifications we could make, we would like very much to modify them and we really appreciate your help.

Sincerely,

Longji Li

1. Apergis N, Poufinas T, Antonopoulos A. ESG scores and cost of debt[J]. Energy Economics, 2022, 112: 106186.
2. Liu Xiangqiang, Yang Qingqing, Hu Jun.ESG rating divergence and Stock price Synchronization [J]. China Soft Science,2023(08):108-120.
